# Inhibition of FOXO3 Tumor Suppressor Function by βTrCP1 through Ubiquitin-Mediated Degradation in a Tumor Mouse Model

**DOI:** 10.1371/journal.pone.0011171

**Published:** 2010-07-02

**Authors:** Wen-Bin Tsai, Young Min Chung, Yiyu Zou, See-Hyoung Park, Zhaohui Xu, Keiko Nakayama, Sue-Hwa Lin, Mickey C-T. Hu

**Affiliations:** 1 Department of Molecular Pathology, University of Texas M. D. Anderson Cancer Center, Houston, Texas, United States of America; 2 Division of Gynecologic Oncology, Stanford University School of Medicine, Stanford, California, United States of America; 3 Department of Medicine, Albert Einstein College of Medicine, New York, New York, United States of America; 4 Department of Pediatrics-Infectious Disease, Baylor College of Medicine, Houston, Texas, United States of America; 5 Division of Developmental Genetics, Center for Translational and Advanced Animal Research on Human Diseases, Tohoku University Graduate School of Medicine, Miyagi, Japan; Garvan Institute of Medical Research, Australia

## Abstract

**Background:**

The ubiquitin-proteasome system is the primary proteolysis machine for controlling protein stability of the majority of regulatory proteins including those that are critical for cancer development. The forkhead box transcription factor FOXO3 plays a key role in regulating tumor suppression; however, the control of FOXO3 protein stability remains to be established. It is crucial to elucidate the molecular mechanisms underlying the ubiquitin-mediated degradation of FOXO3 tumor suppressor.

**Methodology and Principal Findings:**

Here we show that βTrCP1 oncogenic ubiquitin E3-ligase interacts with FOXO3 and induces its ubiquitin-dependent degradation in an IκB kinase-β phosphorylation dependent manner. Silencing βTrCP1 augments FOXO3 protein level, resulting in promoting cellular apoptosis in cancer cells. In animal models, increasing FOXO3 protein level by silencing βTrCP1 suppresses tumorigenesis, whereas decreasing FOXO3 by over-expressing βTrCP1 promotes tumorigenesis and tumor growth *in vivo*.

**Conclusions/Significance:**

This is a unique demonstration that the βTrCP1-mediated FOXO3 degradation plays a crucial role in tumorigenesis. These findings significantly contribute to understanding of the control of FOXO3 stability in cancer cells and may provide opportunities for developing innovative anticancer therapeutic modalities.

## Introduction

FOXO3 (or FOXO3a) is a member of the forkhead box class O (FOXO) transcription factors which have been shown to play critical roles in modulating a number of cellular processes, such as metabolism, differentiation, and transformation in animal cells [1]–[7]. Strikingly, recent gene knockouts reveal FOXOs' vital functions in tumor suppression [8], [9] and the maintenance of the hematopoietic stem cell pool [8], [10], [11]. Activation of FOXO factors was shown to regulate the expression of specific target genes that modulate the cell metabolic state, oxidative stress, aging [1], [2], [12], and those that control cell cycle progression including cyclin B and Polo-like kinase [13], [14] and DNA damage [15]–[17].

While multiple mechanisms have been shown to regulate FOXO3 activity, there is a consensus that nuclear translocation of FOXO3 protein is critical to its regulation and function. Upon stimulation with growth factors, tyrosine kinase receptors trigger phosphoinositide 3-kinase (PI3K) to activate serine/threonine kinases such as the Akt family of protein kinases that phosphorylate FOXO3 protein. Phosphorylated FOXO3 protein then binds to 14-3-3 proteins that facilitate the translocation of FOXO3 from the nucleus into the cytoplasm [1], [2], [18]. This nuclear exclusion and translocation of FOXO3 into the cytoplasm inhibits FOXO3-dependent transcription. In the absence of stimulation from survival signals, Akt is inactivated in quiescent cells, which results in retention of FOXO3 protein in the nucleus and activation of FOXO3-dependent transcription. Other kinases such as IκB kinase (IKK)-**β** and JNK are also important in regulating nuclear exclusion of FOXO3, resulting in inhibition of FOXO3 function. Loss of function of FOXO3 through phosphorylation has been linked to tumorigenesis and poor patient survival in cancer, suggesting that FOXO3 is a key tumor suppressor [3], [5], [8], [19].

In addition to phosphorylation, the protein degradation mechanism plays a key role in regulating FOXO3 tumor suppressor function. The ubiquitin (Ub)-proteasome pathway is critical for regulating degradation of several tumor suppressors such as p53, retinoblastoma, and p27Kip1 proteins in cancer cells and the E3 Ub-ligases important for the Ub-mediated degradation of these proteins have been studied [20], [21]. Interestingly, the FOXO3 protein stability is also regulated by the Ub-proteasome pathway [5], [6], [19]. However, the E3 Ub-ligases necessary for the Ub-mediated degradation of FOXO3 and their mechanisms are not well established. Thus, it is crucial to uncover the E3 Ub ligases required for FOXO3 protein degradation and elucidate their molecular mechanisms. Beta-transducin repeat-containing protein (βTrCP), a novel WD protein [22]–[26], is a unique E3 Ub-ligase that plays a major role in substrate recognition in certain Ub-proteasome pathways [26]. It has been shown that βTrCP1 (also called Fbw1a or FWD1, etc.) ubiquitinates substrates such as phosphorylated IκB [23] and β-catenin [25] and regulates various signaling pathways, which are important for tumorigenesis [26].

Here we show that βTrCP1 is a key E3 Ub-ligase that regulates the protein degradation of FOXO3 tumor suppressor. βTrCP1 interacts with FOXO3 and induces its Ub-dependent degradation in an IKKβ–phosphorylation dependent manner. Using animal models, we further show that downregulation of FOXO3 protein level by ectopic expression of βTrCP1 in breast cancer cells promotes tumor proliferation or tumorigenesis. In contrast, upregulation of FOXO3 protein level by knocking down the expression of βTrCP1 in breast cancer cells suppresses their tumor growth *in vivo*. These results suggest that βTrCP1 may inhibit FOXO3 activity through a novel Ub-mediated degradation mechanism. The important biological and pathological significance of this mechanism in the development of cancer is discussed.

## Results and Discussion

While we examined the expression of FOXO3 protein in various cancer cell lines, we unexpectedly found that the level of FOXO3 protein appeared to be inversely correlated with the level of βTrCP1 protein in various cancer cell lines (not shown), suggesting that βTrCP1 E3 Ub-ligase may contribute to a decrease of FOXO3. Consistently, over-expression of βTrCP1 reduced FOXO3 protein in 293T cells and this reduction could be reverted by treating cells with proteasome inhibitors such as MG-132 or clastro-lactacystin (Figure S1A), suggesting that βTrCP1 may decrease FOXO3 protein level through proteasomal degradation. Additionally, over-expression of mutant βTrCP1ΔF-myc, which contains a deletion at the N-terminal F-box domain (a.a. 17-52) that abolishes its E3 ligase activity [26], increased the protein level of FOXO3 as compared to that in cells cotransfected with the FOXO3-Flag and βTrCP1-myc in the absence of proteasome inhibitor MG-132 (Figure S1B), suggesting that the expression of βTrCP1ΔF may inhibit the degradation of FOXO3 protein mediated by endogenous βTrCP1.

**Figure 1:**
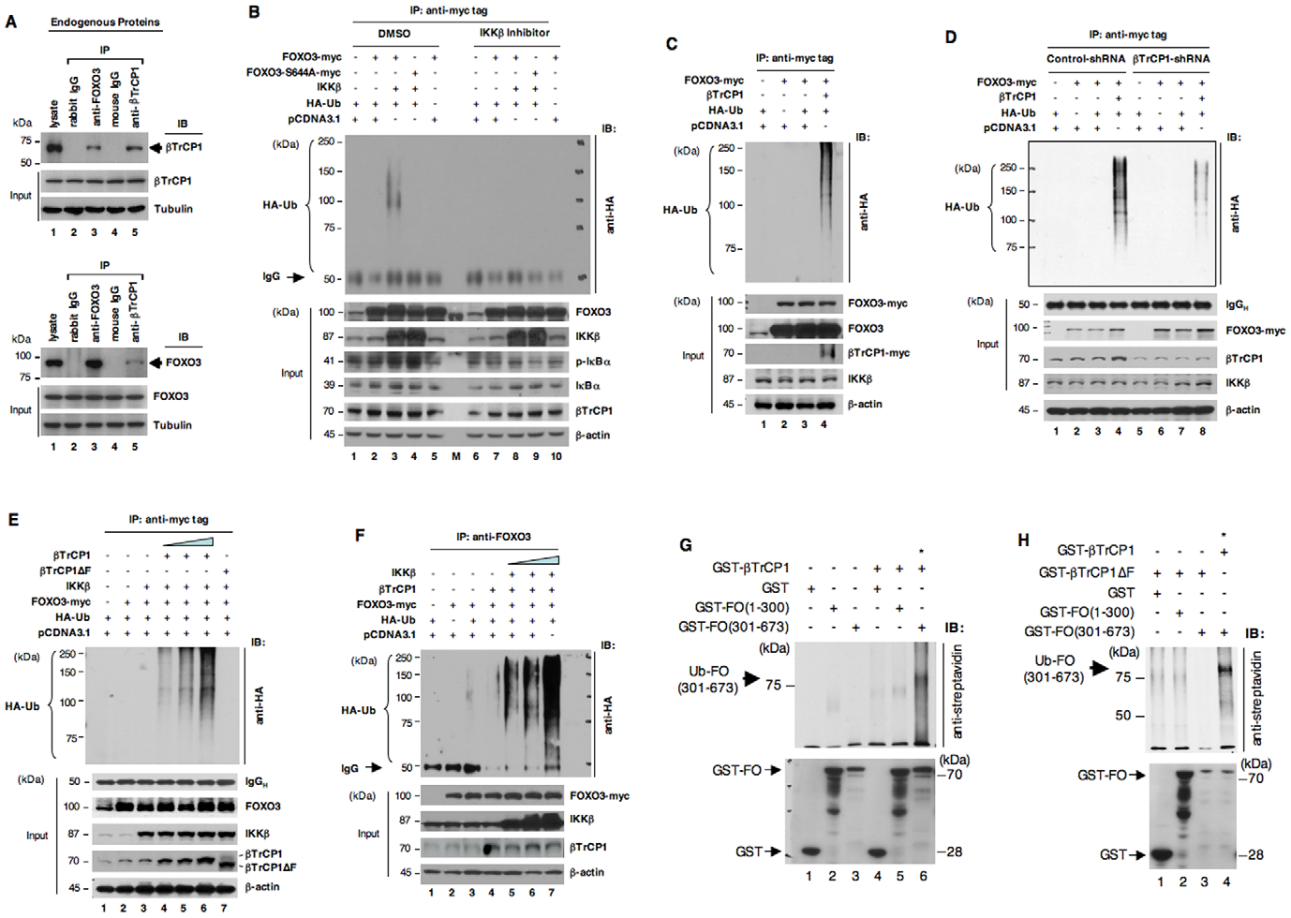
βTrCP1 protein interacts with FOXO3 protein and induces its ubiquitination *in vivo* and *in vitro* in an IKKβ–phosphorylation dependent manner. (**A**) Endogenous proteins in total lysates of MCF-7 cells were subjected to IP with an antibody (Ab) as indicated followed by immunoblotting (IB) with an anti-βTrCP1 or anti-FOXO3 Ab. A rabbit IgG or an isotype mouse IgG were included as an IP negative control. The input IB data indicated the integrity of lysates used for IP. (**B**) 293T cells were cotransfected with HA-ubiquitin (Ub) and FOXO3-Myc or FOXO3-S644A expression vectors plus IKKβ or a control vector (pCDNA3.1) as indicated. At 24 hours after transfection, cells were treated with either DMSO (control) or the IKKβ inhibitor (2 µM) plus the proteasome inhibitor MG-132 (10 µM) for 5 hours. Total lysates were prepared and sonicated extensively for preventing non-covalent protein-protein associations, and subjected to IP with an anti-myc Ab and followed by IB analysis with an anti-HA Ab. The typical pattern of HA-tagged Ub was highlighted. The input IB data were performed with Abs against FOXO3, IKKβ, phospho-IκBα (p-IκBα), IκBα, βTrCP1, and β-actin to show the expressions of transfected vectors in cells as indicated, the effect of the IKKβ inhibitor (as indicated by the repression of p-IκBα level), and the integrity of each protein in total lysates used for IP. (**C**) IP was performed with the lysates of 293T cells that were cotransfected with the expression vectors as indicated, treated with MG-132 as described above, and followed by IB with an anti-HA Ab. The input IB data were performed with Abs against myc-tag, FOXO3, βTrCP1, IKKβ, and β-actin to show the expressions of transfected vectors in cells and the integrity of lysates used for IP. (**D**) Firstly, 293T cells were transfected with either control-shRNA or βTrCP1-shRNA vectors. Twenty four hours post transfection, these cells were cotransfected with the expression vectors as indicated. Then, at 24 hours after cotransfection, cells were treated with MG-132 (20 µM) for 4 hours. The total lysates of these 293T cells were prepared and IP was performed as described above and followed by IB with an anti-HA Ab. The input IB data were performed with Abs against myc-tag, βTrCP1, IKKβ, and β-actin as described. (**E**) 293T cells were cotransfected with various doses of βTrCP1 expression vector and mutant βTrCP1ΔF vector and other expression vectors as indicated. IP was performed with the lysates of these transfected 293T cells, treated with MG-132 as described above, and followed by IB with an anti-HA Ab. The input IB data were performed with Abs against FOXO3, IKKβ, βTrCP1, and β-actin to show the expressions of transfected vectors and endogenous proteins in transfected cells and the integrity of lysates used for IP. (**F**) 293T cells were cotransfected with various doses of IKKβ expression vector and other expression vectors as indicated. IP was performed with the lysates of these transfected 293T cells, treated with MG-132, and followed by IB with an anti-HA Ab as described above. The input IB data were performed with the indicated Abs as described above. (**G**) *In vitro* ubiquitination assays. The indicated target proteins GST-FO(1–300), GST-FO(301–673), and GST (negative control) were incubated with or without E3-ligase protein GST-βTrCP1 in Ub buffer containing E1, E2 (UbcH5b), Mg-ATP, biotinylated ubiquitin, and IKKβ and analyzed by SDS-PAGE and IB with streptavidin conjugated with HRP or an anti-GST Ab as protein controls (lower panel). (**H**) The indicated target proteins were incubated with wild-type E3-ligase protein GST-βTrCP1 or mutant GST-βTrCP1ΔF in Ub buffer, and analyzed as described above. The positive signals are highlighted with *.

To determine if βTrCP1 interacts with endogenous FOXO3 *in vivo*, we carried out co-immunoprecipitation (IP) with an antibody (Ab) against FOXO3 followed by immunoblotting (IB) analysis with an Ab against βTrCP1 or a reciprocal co-IP with an Ab against βTrCP1 followed by IB analysis with an anti-FOXO3 Ab. Indeed, endogenous FOXO3 protein was associated with βTrCP1 protein *in vivo* (Figure 1A). Using the co-transfection approach, we further confirmed that HA-FOXO3 protein was specifically associated with myc-βTrCP1 protein in the transfected cells (Figure S2A,B).

Because the FOXO3 protein is targeted for proteasomal degradation after its phosphorylation in mammalian cells [5], [6], [19], we sought to determine whether phosphorylation of FOXO3 by IKKβ affects the protein level and ubiquitination (Ub) status of FOXO3. Indeed, Ub of FOXO3 protein was enhanced significantly when FOXO3a-myc was co-transfected with HA-Ub and IKKβ in 293T cells in the absence of the IKKβ inhibitor (Figure 1B). Ub of FOXO3 was abrogated by a point mutation at Ser-644 (FOXO3-S644A-myc, whose Ser residue at 644 was mutated to Ala and resistant to IKKβ phosphorylation), suggesting that Ub of FOXO3 requires phosphorylation of FOXO3 at Ser-644 by IKKβ. To demonstrate the requirement for IKKβ activity in the Ub of FOXO3, we did experiments with the IKKβ inhibitor and showed that inhibition of IKKβ activity abrogated the Ub of FOXO3 in 293T cells (Figure 1B), suggesting that the Ub of FOXO3 is dependent on IKKβ activity. As controls, we showed the endogenous βTrCP1 expression in 293T cells by IB analysis (Figure 1B), suggesting that endogenous βTrCP1 is sufficient to facilitate IKKβ-mdeiated the Ub of FOXO3 in 293T cells. In addition, we showed that the IKKβ inhibitor significantly repressed IKKβ activity in phosphorylation of its cellular substrate IκB and overrode the IKKβ-mediated FOXO3 degradation in the treated cells (Figure S3A-C). To confirm if over-expression of IKKβ induces FOXO3 protein degradation, we showed that co-transfection of FOXO3 with IKKβ into 293T cells resulted in reduction of the protein level of FOXO3 and this reducing effect could be overridden by treating cells with various proteasome inhibitors (Figure S2C), suggesting that IKKβ mediates proteasomal degradation of FOXO3 protein.

To determine whether βTrCP1 regulates the Ub status of FOXO3, we showed that Ub of FOXO3 was enhanced significantly when FOXO3-myc was co-transfected with HA-Ub plus βTrCP1 in 293T cells (Figure 1C), suggesting that βTrCP1 promotes Ub of FOXO3 *in vivo*. As controls, we showed the endogenous IKKβ expression in 293T cells by IB analysis (Figure 1C), suggesting that endogenous IKKβ is sufficient to facilitate the βTrCP1-induced Ub of FOXO3 in 293T cells. To demonstrate the requirement for βTrCP1 protein in the Ub of FOXO3, we did experiments with and without silencing of βTrCP1 by using βTrCP1-shRNA in the cotransfected 293T cells and showed that knockdown of βTrCP1 significantly attenuated the Ub of FOXO3 in these cells (Figure 1D), suggesting that the Ub of FOXO3 is dependent of βTrCP1 protein.

To titrate in transfected βTrCP1 and IKKβ to determine whether there are synergistic effects of these proteins on FOXO3 Ub, we showed that increasing in transfected βTrCP1 indeed enhanced the Ub of FOXO3 proportionally in cells cotransfected both βTrCP1 and IKKβ (Figure 1E). Similarly, we demonstrated that increasing in transfected IKKβ augmented the Ub of FOXO3 correspondingly in cells cotransfected both IKKβ and βTrCP1 (Figure 1F). These results imply that there may be an additive or synergistic effect of both proteins (IKKβ and βTrCP1) on FOXO3 Ub. However, we do not have definitive evidence to prove that the observed increase of FOXO3 Ub is due to synergistic effects of these proteins on FOXO3 Ub.

To show direct Ub of FOXO3 by βTrCP1 E3 Ub-ligase, we performed the *in vitro* Ub reactions and showed that βTrCP1 induced Ub of FOXO3 protein *in vitro* (Figure 1G). Although over-expression of the mutant βTrCP1ΔF did not alter its binding to FOXO3 significantly (Figure S2A,B), Ub and degradation of FOXO3 protein were abrogated in the reactions using βTrCP1ΔF defective in E3 activity (Figure 1H; Figure S1B), suggesting that Ub-mediated degradation of FOXO3 protein is directly consequential to the E3 activity of βTrCP1.

**Figure 2:**
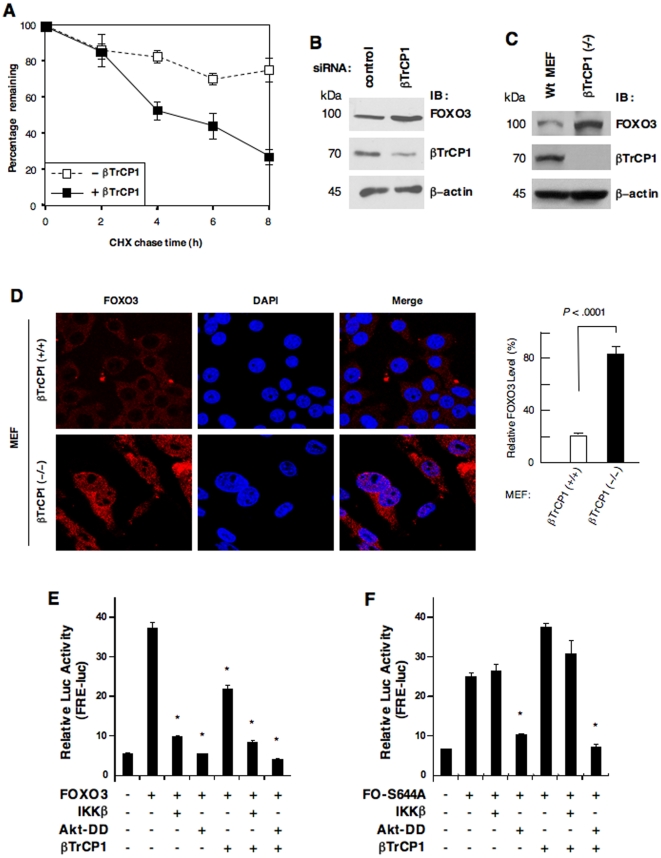
βTrCP1 induces degradation of FOXO3 protein and inhibits its transactivating activity. (**A**) The stability of FOXO3 protein was assayed by cycloheximide (CHX) chase in 293T cells co-transfected with FOXO3-myc and βTrCP1 or a control vector (pCDNA3.1). Lysates of the transfected cells were prepared at 2, 4, 6, 8 hour (h) and 0 h (control) after addition of CHX, and subjected to immunoblotting (IB) with an anti-myc Ab. Graph shows results of densitometric analysis of 3 CHX chase experiments (mean ± SD) using 0 h (control) as 100%. (**B**) MCF-7 cells were transfected with small interfering RNA (siRNA) targeting βTrCP1 or control-siRNA (control). Total lysates of the transfected cells were subjected to IB analysis with an Ab against βTrCP1 or FOXO3 or β-actin (loading control) as indicated. (**C**) Total lysates of wild-type (Wt) and βTrCP1(–/–) MEF cells were subjected to IB analysis with an Ab against FOXO3 or βTrCP1 or β-actin (loading control). (**D**) βTrCP1(–/–) and βTrCP1(+/+) MEF cells were fixed, and the expression and subcellular localization of endogenous FOXO3 was detected using an anti-FOXO3 Ab and followed by an Alexa Fluor 546 (red) -conjugated secondary Ab, and analyzed with fluorescence microscopy. A fluorescent dye 4′-6-Diamidino-2-phenylindole (DAPI) was used to visualize the nuclei. An average of ∼200 cells stained with anti-FOXO3 Ab were analyzed and a histogram shows the relative FOXO3 expression level in MEF cells. (**E** and **F**) Total lysates of 293T cells cotransfected with FRE-luc (firefly luciferase (luc) reporter containing FOXO-responsive elements), pRL-TK (renilla luc as a transfection control for normalization), βTrCP1, FOXO3 (A) or FOXO3-S644A (FO-S644A) (B), plus IKKβ or Akt-DD (an active Akt) as indicated and subjected to luc assays.

To confirm that the Ub–mediated FOXO3 protein degradation is mediated through βTrCP1, we compared the stability of FOXO3 protein in cells co-transfected with βTrCP1. Over-expression of βTrCP1 resulted in a rapid decrease in FOXO3 protein level (Figure 2A; Figure S4A), suggesting that βTrCP1 plays a crucial role in the degradation of FOXO3. We estimated that the half-life of FOXO3 is around 4 hours in cells over-expressing βTrCP1. In addition, silencing βTrCP1 in MCF-7 cells markedly increased FOXO3 protein (Figure 2B). Furthermore, mouse embryonic fibroblasts (MEF) lacking βTrCP1 also augmented the expression of FOXO3 protein (Figure 2C,D), suggesting that βTrCP1 may be essential for the Ub–mediated degradation of FOXO3.

As controls, we showed that MCF-7 and MEF cells express similar levels of IKKβ protein (Figure S4B-D). It should be noted that FOXO3 does not seem to be degraded by endogenous βTrCP1 rapidly in 293T cells under normal conditions. Because the activity of IKKβ is usually regulated by certain inflammatory cytokines such as tumor necrosis factor-α or other oncogenic factors, it is possible that FOXO3 is not subjected to fast degradation in the absence of overexpressed βTrCP1 due to the absence of constitutive phosphorylation of FOXO3 by IKKβ under normal conditions.

Next, we sought to determine whether the βTrCP1–promoted, IKKβ–dependent, degradation of FOXO3 affects its transactivational activity. Using the reporter co-transfection assays, we showed that ectopic expression of βTrCP1 resulted in a significant (∼40%) decrease in FOXO3 transactivational activity (Figure 2E). The mutation of FOXO3 at Ser-644 abrogated the inhibitory effect of IKKβ on the activity of FOXO3 while expression of Akt-DD resulted in a marked decrease in the activity of FOXO3-S644A (Figure 2F). In contrast to the wild-type (wt) FOXO3, over-expression of βTrCP1 could not facilitate the IKKβ–mediated inhibition of mutant FOXO3-S644A.

**Figure 3:**
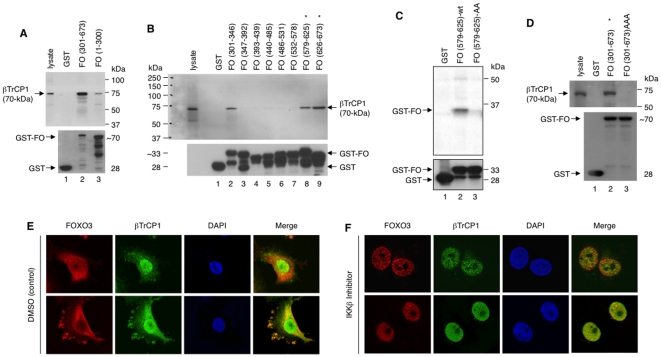
FOXO3 protein is associated and co-localized with βTrCP1 protein and inhibition of IKKβ abolishes co-localization of FOXO3 with βTrCP1 in cells. (**A**) GST-pull down *in vitro* assays. Whole lysates from 293T cells overexpression of IKKβ were incubated with the GST-FOXO3 [GST-FO (1–300) and GST-FO (301–673)] fusion proteins as indicated and GST alone (negative control), and analyzed by SDS-PAGE and immunoblotting (IB) with an anti-βTrCP1 Ab (upper panel) and an anti-GST Ab as protein controls (lower panel). (**B**) The same lysates were incubated with the eight GST-FO sequential fusion fragments spanning the entire carboxy (C)-terminal region of FOXO3 (301–673) as indicated and GST, and analyzed as described. (**C**) *In vitro* kinase assays. Lysates of 293T transfected with HA-IKKβ were IP with an anti-HA, and kinase assays were performed with fusion proteins GST-FO (579–625) and GST-FO (579–625)-AA, whose Ser-586 and Ser-590 residues were mutated to Ala (A) residues, and GST protein (negative control). (**D**) Lysates from 293T cells were incubated with fusion proteins GST-FO (301–673) and GST-FO (301–673)AAA, whose Ser-586, Ser-590, and Ser-644 residues were mutated to Ala residues in the C-terminal domain of FOXO3, and GST protein, and analyzed by IB with an anti-βTrCP1 Ab (upper panel) and an anti-GST Ab (lower panel). The significant signal is indicated with *. (**E**) MCF-7 cells were treated with DMSO (control vehicle for the IKKβ inhibitor shown in **F** below) or untreated, fixed, and the subcellular localizations and co-localization of endogenous FOXO3 and βTrCP1 proteins were detected using an anti-FOXO3 and a specific anti-βTrCP1 Ab (obtained from K. Strebel at NIH) [22] and followed by an Alexa Fluor 594- or 488-conjugated secondary Ab, respectively, and fluorescence microscopy. DAPI was used to show the nuclei, and co-localization of FOXO3 with βTrCP1 was shown as the merged images (yellow). These results are the same as those untreated cells. (**F**) MCF-7 cells were treated with the IKKβ inhibitor (2 µM), fixed, and the subcellular localizations and co-localization of endogenous FOXO3 and βTrCP1 proteins were detected using Abs against FOXO3 and βTrCP1 (NIH) as described above and followed by an Alexa Fluor 594- or 488-conjugated secondary Ab, respectively, and fluorescence microscopy. DAPI was used to show the nuclei, and no co-localization (the merged yellow images) between nuclear FOXO3 and cytoplasmic βTrCP1 was detected.

To elucidate the biochemical mechanism underlying the βTrCP1-mediated degradation of FOXO3, we examined the binding between FOXO3 and βTrCP1 by using GST pull-down assays. We found that βTrCP1 bound FOXO3 mainly at the C-terminal portion [amino acid (a.a.) 301-673] (Figure 3A). Further mapping showed that βTrCP1 bound FOXO3 primarily at the C-terminal domain (a.a. 626–673) and, to a lesser extent, at an adjacent domain (a.a. 579–625) and a distant domain (a.a. 301–346) of FOXO3 (Figure 3B; Figure S5A). In addition to the Ser-644 residue in the C-terminal domain (a.a. 626–673), we identified a new candidate IKKβ phosphorylation motif containing Ser-586 and Ser-590 in the adjacent domain (a.a. 579–625) (Figure S5B). Using *in vitro* kinase assays, we showed that these two Ser residues could be phosphorylated by IKKβ whereas mutation of these two Ser residues to Ala residues abolished phosphorylation by IKKβ (Figure 3C), suggesting that Ser-586 and Ser-590 are indeed new IKKβ phosphorylation sites. Interestingly, mutations of all these sites (Ser-586, -590, and -644) in the C-terminal domain of FOXO3-(301–673) GST fusion protein abrogated the interaction between βTrCP1 and FOXO3-(301–673)AAA (Figure 3D), suggesting that phosphorylation of these sites by IKKβ may be crucial for promoting βTrCP1 binding to FOXO3.

It should be noted that βTrCP1 appears to bind to the 301–346 domain of FOXO3 also. It has been well established that βTrCP1 binds to its substrates through the consensus degron domain “DSGxxS,” where both serines (S) are phosphorylated by kinases [26]. However, the 301–346 domain of FOXO3 does not contain such a motif, suggesting that this binding may be independent of serine phosphorylation and IKKβ-mediated phosphorylation. Although it is interesting that βTrCP1 binds to a site of FOXO3 lacking the degron motif, details of the mechanism underlying the interaction between βTrCP1 and FOXO3 through a phosphorylation-independent manner remain to be elucidated.

Next, we determined the subcellular localizations of βTrCP1 and FOXO3 proteins and whether endogenous FOXO3 was co-localized with βTrCP1 in cancer cells. Using fluorescence microscopy, we showed that βTrCP1 protein is mainly localized in the nucleus of MCF-7 cells without treatment with any proteasome inhibitor by using the specific anti-βTrCP1 polyclonal Ab [22], kindly provided by Klaus Strebel (at NIH). However, βTrCP1 can be clearly detected in the cytoplasm also (Figure 3E). Although endogenous FOXO3 protein was located more in the cytoplasm than nucleus under the same conditions, the majority of FOXO3 was co-localized with βTrCP1 protein in the cytoplasm of MCF-7 cells (Figure 3E). In contrast, the treatment with the IKKβ inhibitor, βTrCP1 is predominantly detected in the nucleus with this anti-βTrCP1 (NIH) Ab and FOXO3 is also primarily localized in the nucleus (Figure 3F). In addition, we have provided data showing similar subcellular localization of endogenous βTrCP1 in BT-549 cells using this specific anti-βTrCP1 Ab (Figure S6A).

Since it has been shown that βTrCP1 is mainly localized in the nucleus [22]–[26], we sought to rule out the possibility that the observed partial cytoplasmic localization of βTrCP1 may be due to an antibody artifact. To confirm subcellular localization of overexpressed βTrCP1-myc (myc tagged) in different cell types, we transfected MCF-7 and 293T cells with the βTrCP1-myc expression vector and performed immuno-fluorescence (IF) analysis with an anti-myc tag Ab and this specific anti-βTrCP1 (NIH) Ab. In agreement with the previous results, our new data showed that the IF images of both anti-myc and anti-βTrCP1 (NIH) Abs are colocalized mainly in the nucleus but they can be detected in the cytoplasm, at a relatively lower level, reproducibly (Figure S6B,C), suggesting that some βTrCP1 may be truly localized in the cytoplasm under normal conditions. In fact, these results are consistent with the previously published reports [24], [25].

Recently, multiple isoforms of βTrCP1 and βTrCP2 derived from alternative splicing have been found to display differential activities in the regulation of Wnt signaling in MCF-7 cells [27], where βTrCP1-mediated degradation of β-catenin mainly occurs in the cytoplasm [25]. Interestingly, some isoforms of βTrCP1 are localized in both cytoplasm and nuclei (i.e., isoforms “f” and “o” [27]). As most anti-βTrCP1 polyclonal Abs cannot distinguish those isoforms of βTrCP1, it is plausible that some βTrCP1 proteins detected in the cytoplasm by the specific anti-βTrCP1 Ab may represent certain isoforms such as “f” and “o” of βTrCP1.

In agreement with previous reports [1]–[3], [5], [19], phosphoryated FOXO3 is mainly localized in the cytoplasm. Using IF analysis, we further confirm that serine (Ser) residues of FOXO3 proteins were phosphoryated in 293T and MCF-7 cells cotransfected with FOXO3-HA (or FOXO3-myc) plus IKKβ vectors and primarily localized in the cytoplasm (Figure S7B,D), whereas the mutant FOXO3-S644A protein was predominantly localized in the nucleus even in cells overexpressed IKKβ (Figure S8A). As controls, we showed that mutant FOXO3-S644A protein was also primarily localized in the nucleus in cells without IKKβ overexpression (Figure S8B). In contrast, we showed that wt FOXO3-HA protein was primarily colocalized with IKKβ in the cytoplasm of cells overexpressed IKKβ (Figure S8C) while wt FOXO3-HA could be detected in the cytoplasm and nucleus without IKKβ overexpression (Figure S8D).

As cells express a homolog of βTrCP1 (βTrCP2), we examined if βTrCP2 protein could interact with FOXO3 protein *in vitro* and *in vivo*. Using GST pull-down assays, we showed that βTrCP2 protein could not bind to FOXO3 protein directly *in vitro* (Figure S9A). Using IF analysis, we showed that βTrCP2 protein could be detected in both the cytoplasm and nucleus while FOXO3 protein was primarily located in the cytoplasm (Figure S9B). Although it appears that βTrCP2 may be localized somewhat more in the nucleus than cytoplasm, it is possible that some isoforms of βTrCP2 [27], as described above, are located more in the nucleus than cytoplasm. Thus, it is probable that FOXO3 protein may not be co-localized with βTrCP2 in cells under normal conditions (Figure S9B). Collectively, these data suggest that the Ub–mediated FOXO3 protein degradation is mainly mediated through βTrCP1 but may not be regulated by βTrCP2. These results further suggest that cells in which βTrCP1 is silenced alone and βTrCP1(−/−) MEFs express high levels of FOXO3 protein.

**Figure 4:**
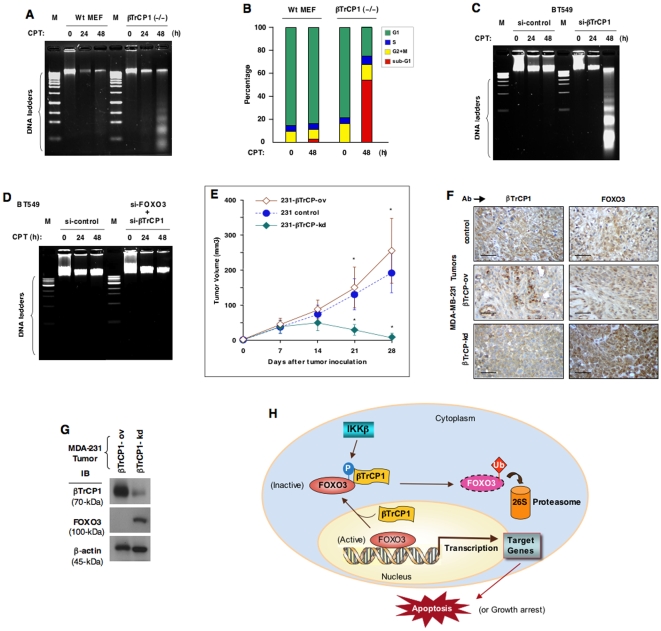
DNA damage induces apoptosis in cells lacking βTrCP1 that can be reverted by silencing FOXO3 and ectopic expression of βTrCP1 promotes tumor growth whereas silencing βTrCP1 in cancer cells suppresses tumorigenesis *in vivo*. (**A**) DNA samples extracted from Wt MEF or βTrCP1(–/–) MEF cells treated with camptothecin (CPT) (20 µM) for 24 or 48 h or control (DMSO) were subjected to DNA fragmentation assay. Equal amounts of the extracted DNA (2 µg/lane) and size markers (M) were subjected to electrophoresis on 2% agarose gels, which were stained with ethidium bromide and photographed. (**B**) MEF cells treated with CPT (20 µM) or DMSO (control) (0 h) for 48 h were stained with propidium iodide (PI), and the cell-cycle profiles were determined by flow cytometry. The changes in percentage of cell-cycle statuses between CPT treatment and control were shown in a histogram. (**C**) BT549 cells were transfected with βTrCP1-siRNA (si-βTrCP1) or control RNA (si-control). DNA fragmentation assay was performed as described above. (**D**) BT549 cells were transfected with control RNA (si-control) alone or transfected with FOXO3-siRNA (si-FOXO3) plus βTrCP1-siRNA (si-βTrCP1). DNA fragmentation assay was performed. (**E**) The 231 cells were transfected with βTrCP1-siRNA (designated 231-βTrCP-kd) or transfected with a βTrCP1 expression vector (designated 231-βTrCP-ov). These transfected cells or 231 control cells were injected into the nude mice as described. *, P<0.05 between 231 control versus 231-βTrCP-kd or 231-βTrCP-ov. (**F**) At 28 days after tumor cell implantation, breast tumors derived from the nude mice bearing 231 (control) or 231-βTrCP-ov or -βTrCP-kd tumors were resected, fixed, sectioned, and placed on slides. Tumor specimens were subjected to immuno-histochemical staining with an Ab specific to βTrCP1 or FOXO3. Slides were examined at 40× magnification with a microscope and representative fields are shown. Scale bars indicate 50 µm. (**G**) Total lysates prepared from 231-βTrCP-ov and 231-βTrCP-kd breast tumor specimens were subjected to IB analysis with Abs against FOXO3, βTrCP1, and β-actin (loading control). (**H**) A diagram depicts the role of βTrCP1 in promoting tumorigenesis through inducing degradation of FOXO3 protein. The subcellular localization of FOXO3 is shown to denote FOXO3 activity.

Moreover, we determined if this increase of FOXO3 protein by silencing βTrCP1 would affect cell survival or death after DNA damage. We showed that exposure of βTrCP1(–/–) MEF cells to camptothecin (CPT), a topoisomerase I inhibitor, for 48 hours led to cellular apoptosis, whereas wt MEFs did not have significant apoptosis (Figure 4A,B). As described above, silencing βTrCP1 increased FOXO3 protein significantly (Figure 2B–D), suggesting that the observed pro-apoptotic phenotype in cells lacking βTrCP1 may be mediated via FOXO3 increase. To further confirm that the apoptotic effect from silencing βTrCP1 was mediated via FOXO3, we showed that silencing both FOXO3 and βTrCP1 together (double knockdown) in BT549 cells abrogated the effect of apoptosis induced by βTrCP1 single knockdown after exposure to CPT (Figure 4C,D), suggesting that the effect of silencing βTrCP1 on the observed apoptosis increase is mediated through FOXO3.

We sought to determine whether silencing endogenous βTrCP1 expression could augment FOXO3 protein level and lead to tumor suppression *in vivo* and whether ectopic expression of βTrCP1 could reduce FOXO3 protein level and promote tumor growth in an orthotopic breast tumor mouse model. When the MDA-MB-231 (abbreviated as 231) cells with βTrCP1-knockdown (231-βTrCP-kd) or 231 control cells were injected into the mammary fat pads of female athymic nude mice, the tumor growth of 231-βTrCP-kd was strongly suppressed as compared to that of 231 control cells (Figure 4E). This tumor suppression phenotype is presumably caused by elevation of FOXO3 that promotes apoptosis. In contrast, when the cells over-expressing βTrCP1 (231-βTrCP-ov) were injected into nude mice in the same fashion, the growth rate of 231-βTrCP-ov cells was enhanced as compared to that of 231 control cells (Figure 4E). This growth increase is presumably induced by the βTrCP1-mediated FOXO3 degradation as described above. Using immunohistochemical staining and IB analysis, we confirmed that the level of FOXO3 was indeed inversely correlated with the expression level of βTrCP1 in the xenograft tumor samples (Figure 4F,G). Because 231 cells are tumorigenic in nude mice and the level of endogenous FOXO3 in 231 cells is low, over-expression of βTrCP1 in 231 cells (231-βTrCP-ov) could only further reduce FOXO3 protein level slightly. Therefore, the tumor growth rate of 231-βTrCP-ov cells in mice could be enhanced only partially as compared to that of 231 control cells (Figure 4E).

We have demonstrated a novel mechanism underlying the Ub-mediated degradation of FOXO3 protein in tumorigenesis. Our findings indicate that βTrCP1 interacts with FOXO3, resulting in promoting Ub-mediated degradation of FOXO3 protein that contributes to tumor growth. This is the first time that βTrCP1 is shown to regulate FOXO3 protein degradation and its tumor suppressor function. Based on these findings, we propose a model for a unique role of βTrCP1 in regulating FOXO3 protein degradation and promoting tumorigenesis (Figure 4H). These findings suggest that cancer cells may acquire sustained resistance to FOXO3-mediated apoptosis through a regulated degradation of FOXO3 protein by βTrCP1 oncogenic E3-ligase. Evidence is emerging to indicate that certain key substrates are targeted by the E3 ligases in the Ub-mediated protein regulation [28]. In addition to FOXO3, it is intriguing that βTrCP1 has been found to interact with another key tumor suppressor p53 [29] and promote its Ub-dependent degradation, resulting in tumorigenesis. Our findings that a unique oncogenic βTrCP1 E3-ligase mediates augmented degradation of FOXO3 protein will significantly contribute to an understanding of the control of FOXO3 protein stability and extend the parallel mechanism of regulation between FOXO3 and p53 in cancer cells. Moreover, this dual inhibition of FOXO3 and p53 tumor suppressor function by βTrCP1 suggests that compounds inhibiting βTrCP1 protein may be innovative therapeutic modalities for suppression of tumor growth and the development of cancer.

## Materials and Methods

### Ethics Statement

All experiments involving mice were approved by the institutional review boards of Stanford University and Albert Einstein College of Medicine. All research involving animals were conducted according to the guidelines of Institutional Animal Care and Use Committees of Stanford University and Albert Einstein College of Medicine. The injected mice were housed in groups, which were provided with food and sterile water. When bedding was contaminated, the mice were transferred into clean cage first and the contaminated bedding was discarded according to Institutional guidelines. There was no change after the appearance of tumors and no nesting material was provided for these mice. The mice were checked daily including weekend.

### Antibodies and reagents

Antibodies (Abs) specific to FOXO3 (FKHRL1, H-144; and N-16), IKKβ, p-IκBα, IκBα, and βTrCP2 were purchased from Santa Cruz Biotechnology (Santa Cruz, CA). A specific anti-FOXO3 Ab was obtained from Imgenex Corp. Abs against β-tubulin, β-actin, hemagglutinin (HA), myc-tag and Flag-tag were purchased from Sigma. An anti-βTrCP1 was obtained from Zymed Laboratories, Inc. (San Francisco, CA). An anti-p27Kip1 Ab was purchased from BD PharMingen (San Diego, CA). Abs against myc-tag (9E10) and poly-ADP-ribose polymerase (PARP) was purchased from Roche Applied Science (Indianapolis, IN), respectively. Additional anti-myc-tag Abs were purchased from Abcam Inc. (Cambridge, MA) and GenScript USA Inc. (Piscataway, NJ). The specific anti-βTrCP1 polyclonal Abs [22] were obtained from K. Strebel (at NIH). Alexa 488 (green)- and Alexa 546 or 555 or 594 or 647 (red)-conjugated secondary Abs and Texas red- and FITC-conjugated secondary Abs were obtained from Molecular Probes (Eugene, OR), Invitrogen Corp. An anti-phosphoserine (p-Ser) Ab was purchased from Invitrogen. Camptothecin (CPT) and the proteasome inhibitors such as MG-132, clastro-Lactacystin (c-Lactacystin), MG-101 (also named ALLN or LLNL), and cycloheximide were purchased from Sigma (St. Louis, MO) or Calbiochem (CA), dissolved in DMSO or ethanol and stored in aliquots at −80°C.

### Cell culture and cell lines

All cell lines were grown under normal conditions at 37°C and 5% CO_2_ in DMEM/F12 supplemented with L-glutamine, penicilline/streptomycin and 10% fetal bovine serum (FBS). The tumor type origins of the cell lines are: MDA-MB-231 and MCF-7, human breast epithelial adenocarcinoma; BT474 and BT549, human breast epithelial ductal carcinoma; 293T, human kidney epithelial cell line expressing SV40 large T antigen. Mouse embryonic fibroblasts (MEF) βTrCP1(–/–) and their control wt MEF cells have been described previously [30].

### Immunoprecipitation (IP) and immunoblotting (IB)

All IP experiments were performed as described previously [17]. Briefly, cells were washed twice with PBS and lysed with lysis buffer containing protease inhibitors at 4°C for 20 min. Total lysates were sonicated extensively for preventing non-specific protein-protein associations. For Ub analysis, the lysates were prepared with lysis buffer under denaturing conditions and sonicated extensively to prevent non-covalent protein-protein associations. Protein samples were first precleared with a nonspecific IgG antibody. Precleared lysates were then incubated with an Ab by rotating at 4°C overnight followed by the addition of 25 ml of 50% protein A- or Protein G-sepharose slurry and rotating for 1 hour. Protein A/G beads were collected and washed with lysis buffer four times. Immunoprecipitates were resolved by 6% or 10% or 12% SDS-polyacrylamide gel eletrophoresis (PAGE) and analyzed by IB analysis. After washing, immunoprecipitates were resolved by SDS-PAGE and analyzed by IB analysis. For IB analysis, the protein samples were subjected to SDS-PAGE and transferred onto nitrocellulose or polyvinylidene difluoride (PVDF) membranes. The membranes were blocked with 5% nonfat dry milk or BSA in PBS containing 0.05% Tween 20 and incubated with primary Abs and then with horseradish peroxidase-conjugated secondary Abs according to the manufacturer's instructions. IB analysis was visualized by an enhanced chemiluminescence (ECL) kit obtained from Pharmacia or Santa Cruz Biotechnology.

### *In vitro* ubiquitination assays

E3-ligase proteins (GST-βTrCP1 and GST-βTrCP1ΔF) and target proteins (GST (negative control), GST-FO(1–300), and GST-FO(301-673)) were produced as GST-fusion proteins from E. coli and purified. In vitro ubiquitination assays were carried out using in vitro ubiquitination assay kit (BIOMOL). In vitro ubiquitination assays were carried out in 50 µl of ubiquitinylation buffer, containing E1 (100 nM), E2 (UbcH5b, 2.5 µM), Mg-ATP (5 mM), E3 (0.1 µM), target protein (1 µM), inorganic pyrophosphatase solution (100 U/ml), DTT (5 mM), Mg-ATP (25 nM), biotinylated ubiquitin (2.5 µM), and the cell extracts (5 µg protein) from 293T cells over-expression of IKKβ. The reaction mixtures were incubated for 2 hours at 37°C and then terminated with 2X nonreducing gel loading buffer. The protein samples were subjected to SDS-PAGE (8% gel) and transferred onto nitrocellulose membrane. The membranes were incubated with streptavidin-HRP solution (Vectastain ABC Elite kit) for 1 hour, washed with BSA/TBST (Tris-buffered saline containing 0.05% Tween 20) solution for 1 hour and visualized by chemiluminescence.

### Immunofluorescence (IF)

MEF βTrCP1(–/–) and βTrCP1(+/+) cells were seeded onto sterile glass coverslips, which were placed in a 12-well culture plate. Cells were fixed with 4% paraformaldehyde for 10 min at room temperature before being permeabilized in 0.5% Triton X-100. Slide culture chambers were washed with PBS and blocked with PBS containing 2% BSA, incubated with an Ab specific to FOXO3 or βTrCP1 (1∶100 to 1∶500 dilution), followed by Alexa 546 or 555 or 594 or 647 (red)-conjugated anti-rabbit and Alexa 488 (green)-conjugated anti-mouse secondary Abs (Molecular Probes, Eugene, OR). Cells were counterstained with DAPI (Molecular Probes, Eugene, OR) to show the nuclei. Specific staining was visualized and images were captured with a Leica SP2 AOBS confocal laser scanning microscope or an Olympus IX81 system confocal microscope as described previously [17].

### siRNA transfection

MCF-7 or 293T cells were transfected with specific small-interfering RNA (siRNA) targeting FOXO3 (si-FOXO3a) (5′-GAGCUCUUGGUGGAUCAUC_d_T_d_T-3′) or βTrCP1 (βTrCP1 siRNA duplexes were obtained from Dharmacon) or control luc siRNA (5′-CUUACGCUGAGUACUUCGA_d_T_d_T-3′) duplex (Dharmacon) (4 uM/2×10^6^ cells) by electroporation using Nucleofector 1 (amaxa) or Lipofectamine 2000 (Invitrogen Corp.) as described previously [17]. The control vector (without shRNA), the scrambled shRNA control vector, and the βTrCP1-shRNAs vectors were purchased from OriGene Technologies, Inc. (Rockville, MD).

### DNA fragmentation Assay

DNA fragmentation in apoptotic cells was determined by the standard gel electrophoresis. Cells were treated with the indicated drugs or control for 24 or 48 hours, harvested, washed with PBS, and incubated with lysis buffer on ice for 20 min. Samples were then centrifuged at 4°C at 12,000×*g* for 30 min. DNA was extracted with phenol/chloroform and precipitated with ethanol. The DNA pellet was then washed with 70% ethanol, dissolved in water containing RNase, and incubated at 37°C for 30 minutes. The extracted DNA (2 µg/lane) was subjected to electrophoresis on 2% agarose gels, stained with ethidium bromide, and then photographed.

### Cell cycle analysis

Wild type (Wt) and βTrCP1(–/–) MEF cells were treated with CPT (20 µM) or control (DMSO) for 5 hours. Cells were rinsed with PBS and fixed in 70% ethanol at 4°C overnight. The fixed cells were then washed twice with PBS and resuspended in PBS containing 10 µg/ml PI (Roche Applied Science) and 10 µg/ml RNase A (Sigma), and incubated for 1 hour at room temperature before analysis. The samples were analyzed by flow cytometry as described previously [17]. Cell populations in different phases of cell cycle were determined.

### GST pull-down assays

For GST pull-down assays: the various GST-fusion FOXO3 plasmids containing the specific domains of FOXO3 (GST-FO-(1–301), (301–673), (301–346), (347–392), (393–439), (440–485), (486–531), (532–578), (579–625), and (626–673)) were prepared. The various GST-fusion FOXO3 expression vectors were constructed as described previously [17]. Briefly, the GST-FO fusion proteins and GST proteins (control) were expressed in *Escherichia coli* BL21 (Invitrogen, Carlsbad, CA) lysed in a GST lysis buffer (50 mM Tris, pH 7.5, 150 mM NaCl, 1% Triton X-100, and protease inhibitors), and immobilized onto glutathione-Sepharose beads (Pharmacia Biotech). Total lysate (1 mg) from 293T cells that were transfected with IKKβ or treated with TNFα (10 ng/ml) in binding buffer (50 mM Tris, pH 7.5, 100 mM NaCl, 10 mM MgCl2, 0.5% Nonidet P-40 and protease inhibitors) was mixed with the GST-FO or GST (control) containing glutathione-Sepharose beads. The protein complex formation on glutathione-Sepharose beads was carried out overnight at room temperature or 4°C with shaking. The beads were washed with binding buffer, and the bound protein complexes were disrupted and proteins were denatured directly by boiling in SDS loading buffer.

### Immunohistochemical staining and statistical analysis

Immunohistochemical staining was performed as described previously [17]. Breast tumors were excised from the tumor-bearing mice 28 days (231-control, 231-βTrCP-ov, and 231-βTrCP-kd tumors) after inoculation of the test or control cells. Five independent tumors (each from a different mouse) were taken from each test group for testing. Tumor samples were fixed in formalin, sectioned, placed on slides, and incubated with specific Abs. Sections were then treated with biotin-conjugated secondary Ab followed by avidin biotin-peroxidase complex and amino-ethyl carbazole as a chromogen. All data are expressed as means and standard deviations (SD) from at least three determinations. The statistical significance of differences in cell proliferation and tumor growth between two groups was analyzed with two-sided unpaired Student's *t* tests when the variances were equal, or with Welch's corrected *t* tests when the variances were unequal, with Graphpad statistical software (San Diego, CA). All statistical tests were two-sided, and P values less than 0.05 were considered statistically significant.

### Animal studies

To determine tumorigenicity and establish orthotopic breast cancer animal models, female athymic (*nu/nu*) nude mice were purchased from the NCI Frederick Cancer Research Center (Frederick, MD) or NCR NU-M-F (Taconic Farm) and maintained aseptically in an athymic animal room. For tumor-cell implantation, cells (derivatives of MDA-MB-231 (231-control, 231-βTrCP1-ov, and 231-βTrCP1-kd)) in log-phase growth were harvested, washed with phosphate buffered saline (PBS), and resuspended in PBS. Then cells (2×10^6^ in 0.1 ml PBS) were injected into the mammary fat pad of each mouse. The tumor sizes were measured twice per week with a Vernier caliper. Data are presented as means and standard deviations of two experiments with 5 mice in each group. All procedures were performed in compliance with guidelines of Institutional Animal Care and Use Committee.

## Supporting Information

Figure S1 Ectopic expression of βTrCP1 decreases FOXO3 protein level that can be reverted by treating cells with proteasome inhibitors. (A) Total lysates from 293T cells that were cotransfected with FOXO3-Flag plus βTrCP1, treated with the proteasome inhibitor MG-132 or clastro-Lactacystin (c-Lactacystin) or DMSO (vehicle control), were analyzed by immunoblotting (IB) with an indicated antibody (Ab). β-actin was used to show the protein loading control. (B) The effect of overexpression of wild-type (wt) and mutant βTrCP1 on the levels of FOXO3 protein. Total lysates of 293T cells that were cotransfected with a control vector (pcDNA3.1) or FOXO3-Flag alone or FOXO3-Flag plus wt βTrCP1-myc or the mutant βTrCP1ΔF-myc (E3 mutant) vector as denoted, treated with MG-132 or DMSO, and untransfected 293T cells (negative control, the far left lane), were analyzed by IB analysis with an indicated Ab as described above. The molecular weights (kDa) of proteins are highlighted.
(3.00 MB TIF)

Figure S2 βTrCP1 is associated with FOXO3 in cells over-expressing these proteins *in vivo*. (A) Total lysates of 293T cells cotransfected with hemagglutinin (HA)-tagged FOXO3 plus a myc-tagged βTrCP1 or mutant βTrCP1ΔF, and IKKβ or an empty vector were analyzed by immuno-precipitation (IP) with an anti-myc tag antibody (Ab) followed by immunoblotting (IB) with an anti-HA Ab. (B) The same lysates as described in A were subjected to reciprocal IP with an anti-HA followed by IB with an anti-myc Ab. IB analysis for FOXO3 or myc-βTrCP or β-actin with the indicated Ab was shown as a control of protein input before IP. (C) Total lysates of 293T cells cotransfected with FOXO3-Flag and IKKβ, treated with c-Lactacystin or MG-101 (also named ALLN or LLNL) or DMSO, were analyzed by IB with an anti-Flag or anti-β-actin Ab (loading control).
(0.15 MB TIF)

Figure S3 The IKKβ inhibitor significantly represses IKKβ activity and overrides the IKKβ-mediated FOXO3 degradation; βTrCP1-shRNAs significantly reduce βTrCP1 expression in transfected cells. (A, B) 293T cells were treated with an equal amount of DMSO (control vehicle) (A) or IKKβ inhibitor (B) under normal cell culture conditions. Four hours after treatment, cells were washed, fixed, and stained with antibodies (Abs) against IKKβ and phospho-IκB (p-IκB) and followed by the Alexa Fluor 488 (green)- and Alexa Fluor 647 (red)-conjugated secondary Abs, respectively, and fluorescence microscopy. A nuclear stain 4′,6-diamidino-2-phenylindole (DAPI) was used to show the nuclei. Co-localizations between IKKβ and p-IκB images are shown as the merged yellow/orange images. (C) Total lysates from 293T cells that were transfected with either IKKβvector or pcDNA3.1 (control) vector, treated with the IKKβ inhibitor or DMSO (control) as indicated, were analyzed by immunoblotting (IB) with an Ab against FOXO3 or IKKβ or p-IκBα or β-actin (as protein loading control). The molecular weights of proteins are highlighted. (D) Over-expression of βTrCP1-shRNAs slienced endogenous βTrCP1 expression in the transfected cells. 293T cells were co-transfected with the control vector (without shRNA) or the scrambled shRNA control vector or the βTrCP1-shRNAs vectors. At 48 hours post transfection, total lysates were prepared from the transfected cells and subjected to IB analysis with an Ab against βTrCP1 or β-actin (loading control) as described above.
(0.72 MB TIF)

Figure S4 Ectopic expression of βTrCP1 increases FOXO3 protein degradation; the test cell lines express similar levels of endogenous IKKβ protein. (A) The 293T cells were cotransfected with FOXO3-myc plus βTrCP1-myc and IKKβ and followed by cycloheximide (CHX) chase (100 µg/ml) at 2, 4, 6, and 8 hour. Total lysates of these cells were analyzed by IB with an anti-myc (detecting FOXO3-myc 100-kDa protein and βTrCP1-myc 70-kDa protein expression control) or anti-β-actin antibody (Ab) (loading control). (B-D) MCF-7 cells (B), mouse embryonic fibroblasts (MEF) βTrCP1(+/+) cells (C), and MEF βTrCP1(−/−) cells (D) were washed, fixed, and stained with an Ab against IKKβ and followed by the Alexa Fluor 488 (green)-conjugated secondary Ab, and fluorescence microscopy. A nuclear stain 4′,6-diamidino-2-phenylindole (DAPI) was used to show the nuclei.
(3.00 MB TIF)

Figure S5 The candidate protein domains and sequence motifs involved in the interaction between FOXO3 and βTrCP1. (A) A diagram depicts the relative positions of GST-FOXO3 (GST-FO) fusion proteins in the entire carboxy-terminal region of FOXO3 (301–673). The orange color highlights a candidate domain in the GST-FO(579–625) or GST-FO(626–673) fragment that interacts with βTrCP1 significantly in the GST-pull down assays. (B) The putative IKKβ consensus sequences for phosphorylation (S, serine; T, threonine; and X, any amino acid) in the FOXO3 (579–625) domain are shown, and the new candidate S residues that are phosphoryated by IKKβ are highlighted by boxes in red dots.
(3.00 MB TIF)

Figure S6 Subcellular localization of βTrCP1 protein in different cell types. (A) BT-549 breast cancer cells were treated with DMSO, fixed, and the subcellular localizations and co-localization of endogenous FOXO3 and βTrCP1 proteins were detected using antibodies (Abs) against FOXO3 and βTrCP1 and followed by an Alexa Fluor 555- or 488-conjugated secondary Abs, respectively, and fluorescence microscopy. DAPI was used to show the nuclei, and co-localizations of FOXO3 with βTrCP1 images are shown as the merged yellow/orange images. (B, C) Overexpression of βTrCP1-myc in MCF-7 cells (B) and 293T cells (C) confirms the subcellular localizations of βTrCP1-myc. At 48 hours after transfection, cells were fixed and stained with Abs against myc-tag or βTrCP1, a specific Ab that was developed by K. Strebel at NIH as described previously [22], and followed by an Alexa Fluor 647- or 488-conjugated secondary Abs, respectively, and fluorescence microscopy as described above.
(3.00 MB TIF)

Figure S7 The phosphorylation status of FOXO3 protein in cells over-expressing FOXO3 and IKKβ. (A, B) 293T cells were co-transfected with FOXO3-HA vector plus control (A) or IKKβ-Flag (B) vector. Forty-eight hours after transfection, cells were washed, fixed, and stained with antibodies against HA-tag and phosphoserine (p-Ser) and followed by the Alexa Fluor 647 (red)- and Alexa Fluor 488 (green)-conjugated secondary antibodies, respectively, and fluorescence microscopy. DAPI was used to show the nuclei. Co-localizations between FOXO3-HA and p-Ser images are shown as the merged yellow/orange images. (C, D) MCF-7 cells were co-transfected with FOXO3-myc vector plus control (C) or IKKβ-Flag (D) vector. Forty-eight hours after transfection, cells were stained with antibodies against myc-tag and p-Ser and followed by secondary antibodies and fluorescence microscopy as described above. Co-localizations between FOXO3-myc and p-Ser images are shown as described.
(3.00 MB TIF)

Figure S8 The subcellular localization of mutant FOXO3-S644A protein in cells overexpressed FOXO3-S644A and IKKβ. (A, B) 293T cells were co-transfected with mutant FOXO3-S644A-myc plus IKKβ-Flag (A) vectors or FOXO3-S644A-myc alone (B). Forty-eight hours after transfection, cells were washed, fixed, and stained with antibodies (Abs) against myc-tag and Flag-tag and followed by the Alexa Fluor 546 (red)- and Alexa Fluor 488 (green)-conjugated secondary Abs, respectively, and fluorescence microscopy. DAPI was used to show the nuclei. (C, D) MCF-7 cells were co-transfected with wild-type FOXO3-HA plus IKKβ-Flag vectors (C) or FOXO3-HA alone (D). At 48 hours post transfection, cells were fixed and stained with Abs against myc-tag and Flag-tag and followed by secondary Abs and fluorescence microscopy as described above. Co-localizations between FOXO3-HA and IKKβ-Flag images are shown as the merged yellow/orange images.
(3.00 MB TIF)

Figure S9 βTrCP2 does not bind FOXO3 protein significantly *in vitro* and *in vivo*. (A) GST-pull down *in vitro* assays. Whole lysates from 293T cells overexpression of IKKβ were incubated with the GST-FOXO3 [GST-FO (1–300) and GST-FO (301–673)] fusion proteins as indicated and GST alone (negative control), and analyzed by SDS-PAGE and immunoblotting with an anti-βTrCP2 antibody (Ab) (upper panel) and an anti-GST Ab (lower panel) as protein controls. (B) Co-localization between endogenous βTrCP2 and FOXO3 in HeLa cells. Cells cultured under normal conditions were stained with Abs against FOXO3 and βTrCP2 and followed by an Alexa Fluor 594 (red)- and Alexa Fluor 488 (green)-conjugated secondary Abs, respectively, and fluorescence microscopy. DAPI was used to show the nuclei. No significant co-localization (the merged yellow images) between endogenous FOXO3 with βTrCP2 was detected.
(3.00 MB TIF)
