# Supplementary figures and images for: Inhibition of FOXO3 Tumor Suppressor Function by βTrCP1 through Ubiquitin-Mediated Degradation in a Tumor Mouse Model
Source: PLoS One. 2010 Jul 2;5(7):e11171. doi: 10.1371/journal.pone.0011171 (PMC2896402; doi:10.1371/journal.pone.0011171)

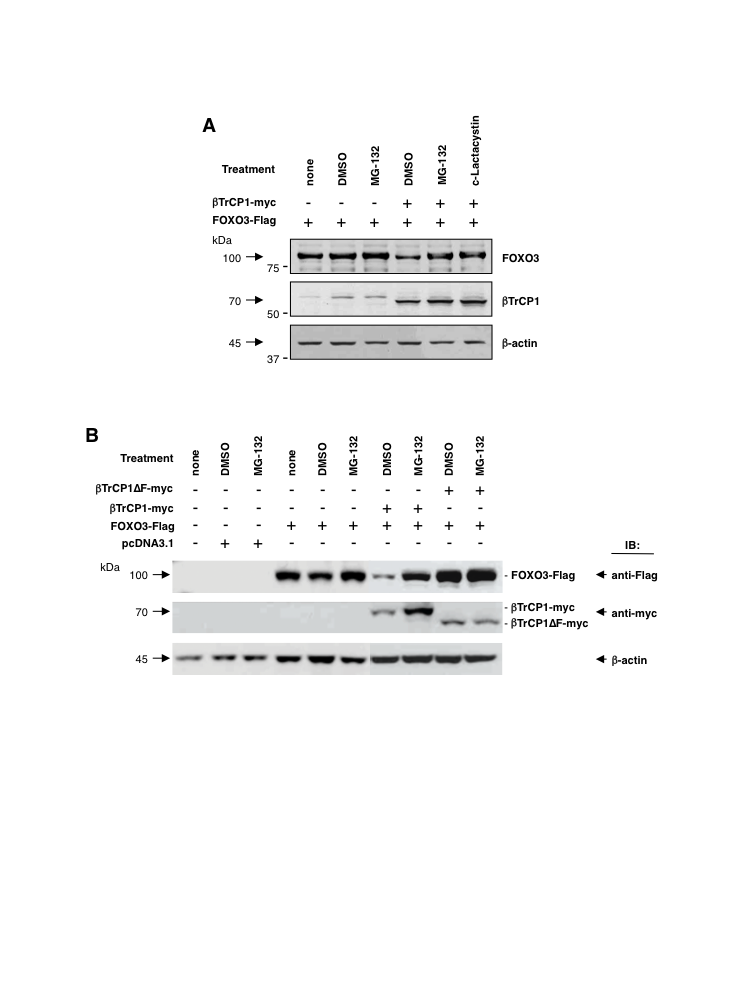

Supplement: Figure S1 — Ectopic expression of βTrCP1 decreases FOXO3 protein level that can be reverted by treating cells with proteasome inhibitors. (A) Total lysates from 293T cells that were cotransfected with FOXO3-Flag plus βTrCP1, treated with the proteasome inhibitor MG-132 or clastro-Lactacystin (c-Lactacystin) or DMSO (vehicle control), were analyzed by immunoblotting (IB) with an indicated antibody (Ab). β-actin was used to show the protein loading control. (B) The effect of overexpression of wild-type (wt) and mutant βTrCP1 on the levels of FOXO3 protein. Total lysates of 293T cells that were cotransfected with a control vector (pcDNA3.1) or FOXO3-Flag alone or FOXO3-Flag plus wt βTrCP1-myc or the mutant βTrCP1ΔF-myc (E3 mutant) vector as denoted, treated with MG-132 or DMSO, and untransfected 293T cells (negative control, the far left lane), were analyzed by IB analysis with an indicated Ab as described above. The molecular weights (kDa) of proteins are highlighted. (3.00 MB TIF) [file pone.0011171.s001.tif]

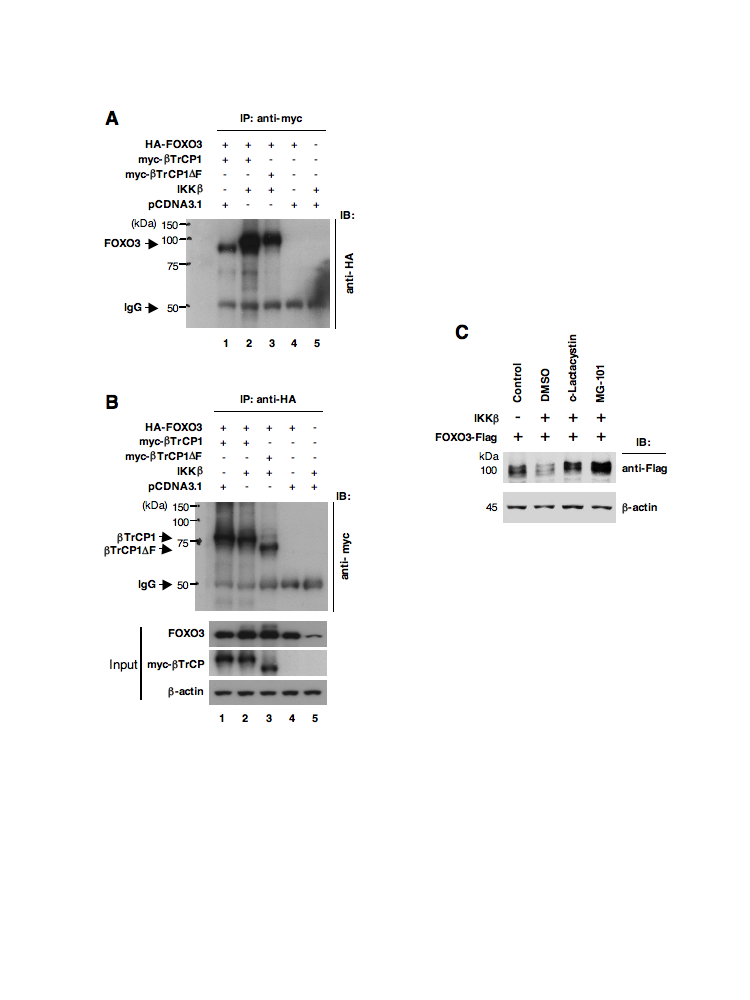

Supplement: Figure S2 — βTrCP1 is associated with FOXO3 in cells over-expressing these proteins in vivo. (A) Total lysates of 293T cells cotransfected with hemagglutinin (HA)-tagged FOXO3 plus a myc-tagged βTrCP1 or mutant βTrCP1ΔF, and IKKβ or an empty vector were analyzed by immuno-precipitation (IP) with an anti-myc tag antibody (Ab) followed by immunoblotting (IB) with an anti-HA Ab. (B) The same lysates as described in A were subjected to reciprocal IP with an anti-HA followed by IB with an anti-myc Ab. IB analysis for FOXO3 or myc-βTrCP or β-actin with the indicated Ab was shown as a control of protein input before IP. (C) Total lysates of 293T cells cotransfected with FOXO3-Flag and IKKβ, treated with c-Lactacystin or MG-101 (also named ALLN or LLNL) or DMSO, were analyzed by IB with an anti-Flag or anti-β-actin Ab (loading control). (0.15 MB TIF) [file pone.0011171.s002.tif]

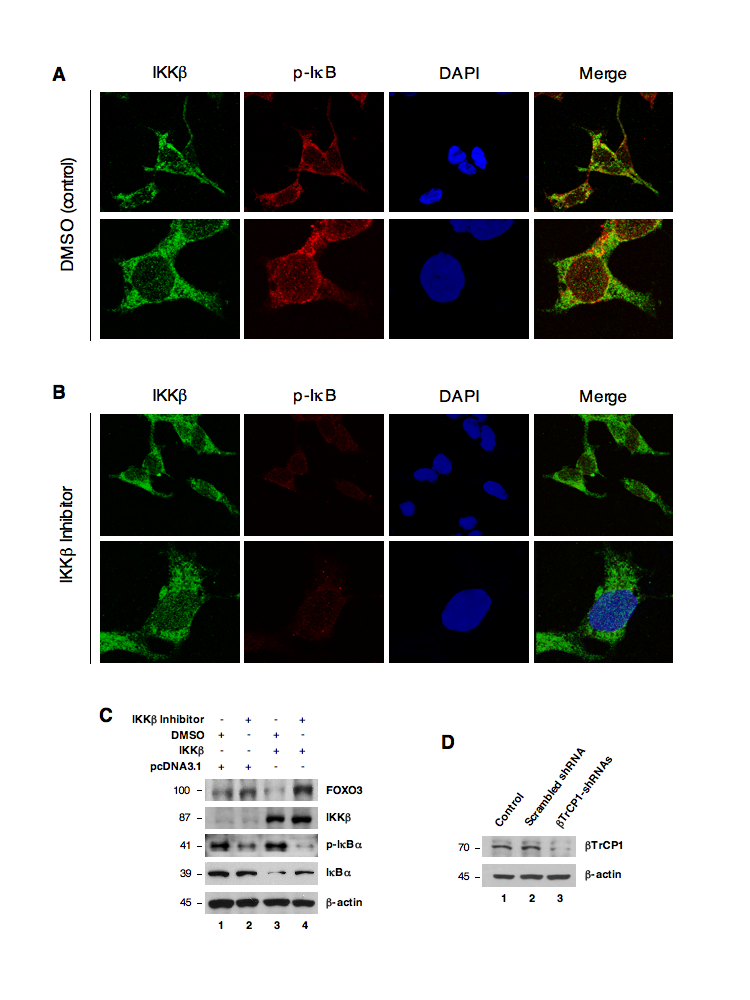

Supplement: Figure S3 — The IKKβ inhibitor significantly represses IKKβ activity and overrides the IKKβ-mediated FOXO3 degradation; βTrCP1-shRNAs significantly reduce βTrCP1 expression in transfected cells. (A, B) 293T cells were treated with an equal amount of DMSO (control vehicle) (A) or IKKβ inhibitor (B) under normal cell culture conditions. Four hours after treatment, cells were washed, fixed, and stained with antibodies (Abs) against IKKβ and phospho-IκB (p-IκB) and followed by the Alexa Fluor 488 (green)- and Alexa Fluor 647 (red)-conjugated secondary Abs, respectively, and fluorescence microscopy. A nuclear stain 4′,6-diamidino-2-phenylindole (DAPI) was used to show the nuclei. Co-localizations between IKKβ and p-IκB images are shown as the merged yellow/orange images. (C) Total lysates from 293T cells that were transfected with either IKKβvector or pcDNA3.1 (control) vector, treated with the IKKβ inhibitor or DMSO (control) as indicated, were analyzed by immunoblotting (IB) with an Ab against FOXO3 or IKKβ or p-IκBα or β-actin (as protein loading control). The molecular weights of proteins are highlighted. (D) Over-expression of βTrCP1-shRNAs slienced endogenous βTrCP1 expression in the transfected cells. 293T cells were co-transfected with the control vector (without shRNA) or the scrambled shRNA control vector or the βTrCP1-shRNAs vectors. At 48 hours post transfection, total lysates were prepared from the transfected cells and subjected to IB analysis with an Ab against βTrCP1 or β-actin (loading control) as described above. (0.72 MB TIF) [file pone.0011171.s003.tif]

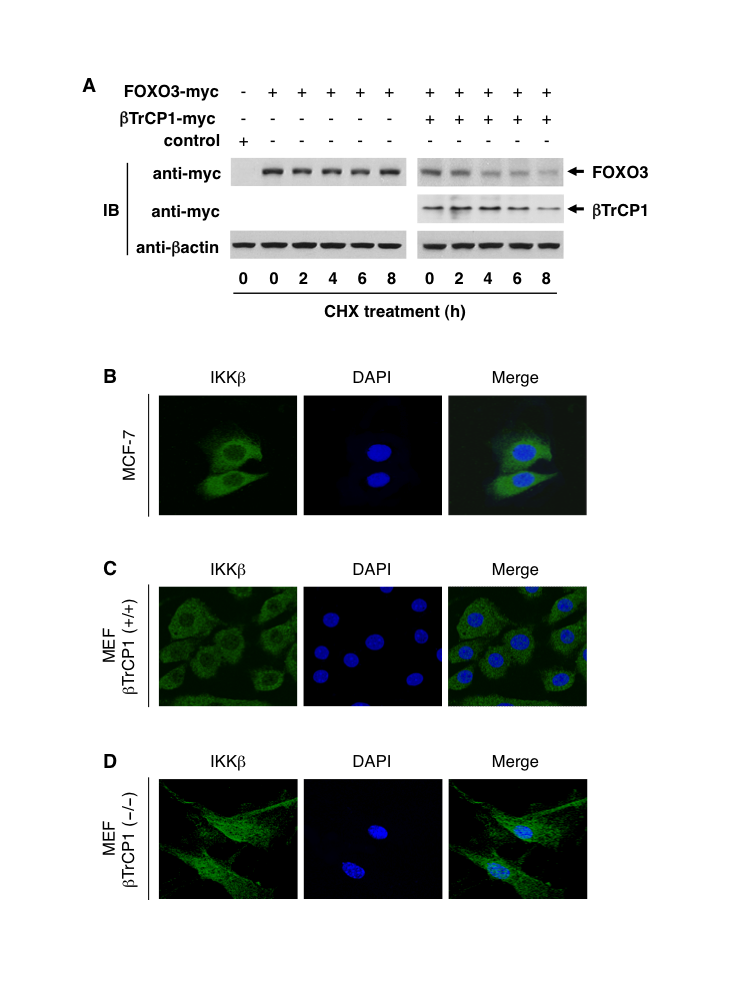

Supplement: Figure S4 — Ectopic expression of βTrCP1 increases FOXO3 protein degradation; the test cell lines express similar levels of endogenous IKKβ protein. (A) The 293T cells were cotransfected with FOXO3-myc plus βTrCP1-myc and IKKβ and followed by cycloheximide (CHX) chase (100 µg/ml) at 2, 4, 6, and 8 hour. Total lysates of these cells were analyzed by IB with an anti-myc (detecting FOXO3-myc 100-kDa protein and βTrCP1-myc 70-kDa protein expression control) or anti-β-actin antibody (Ab) (loading control). (B-D) MCF-7 cells (B), mouse embryonic fibroblasts (MEF) βTrCP1(+/+) cells (C), and MEF βTrCP1(−/−) cells (D) were washed, fixed, and stained with an Ab against IKKβ and followed by the Alexa Fluor 488 (green)-conjugated secondary Ab, and fluorescence microscopy. A nuclear stain 4′,6-diamidino-2-phenylindole (DAPI) was used to show the nuclei. (3.00 MB TIF) [file pone.0011171.s004.tif]

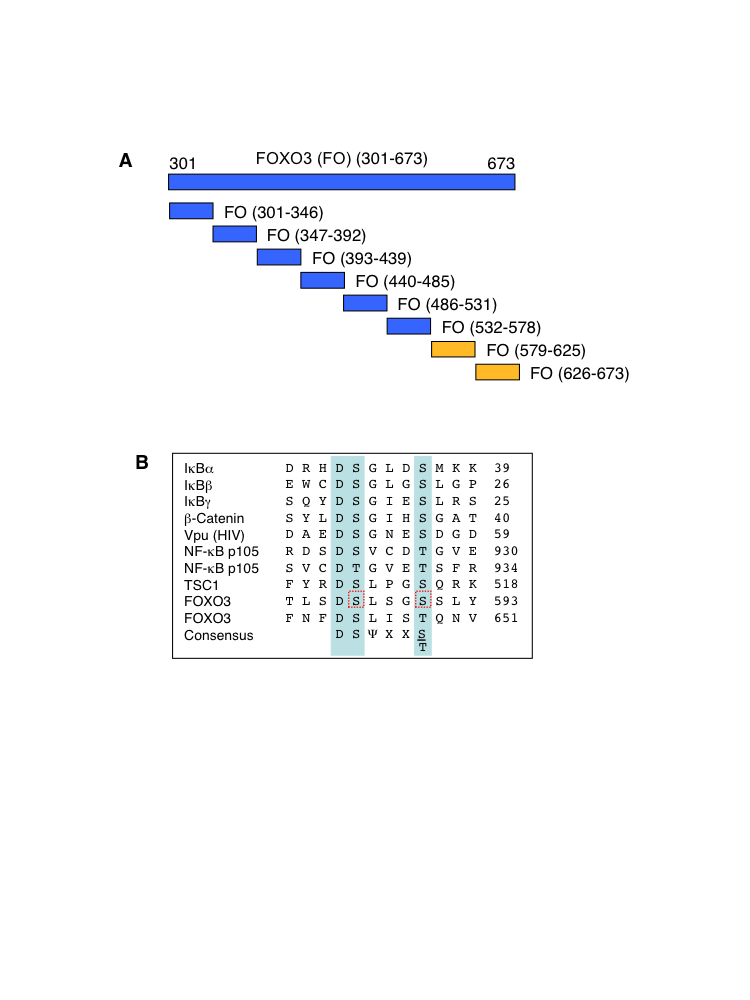

Supplement: Figure S5 — The candidate protein domains and sequence motifs involved in the interaction between FOXO3 and βTrCP1. (A) A diagram depicts the relative positions of GST-FOXO3 (GST-FO) fusion proteins in the entire carboxy-terminal region of FOXO3 (301–673). The orange color highlights a candidate domain in the GST-FO(579–625) or GST-FO(626–673) fragment that interacts with βTrCP1 significantly in the GST-pull down assays. (B) The putative IKKβ consensus sequences for phosphorylation (S, serine; T, threonine; and X, any amino acid) in the FOXO3 (579–625) domain are shown, and the new candidate S residues that are phosphoryated by IKKβ are highlighted by boxes in red dots. (3.00 MB TIF) [file pone.0011171.s005.tif]

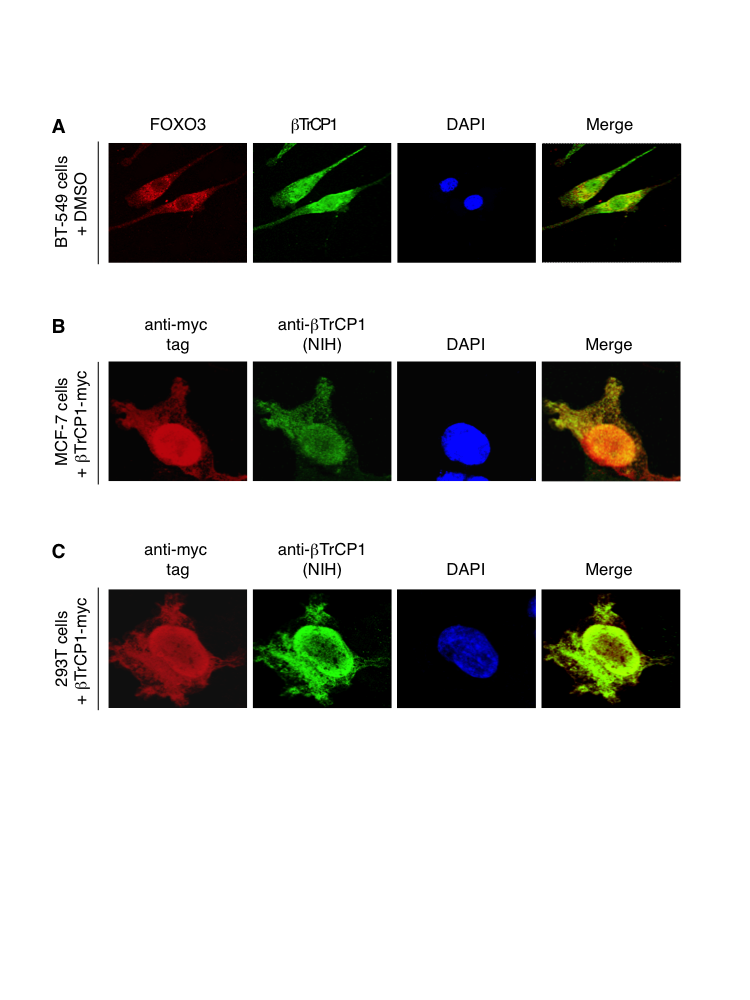

Supplement: Figure S6 — Subcellular localization of βTrCP1 protein in different cell types. (A) BT-549 breast cancer cells were treated with DMSO, fixed, and the subcellular localizations and co-localization of endogenous FOXO3 and βTrCP1 proteins were detected using antibodies (Abs) against FOXO3 and βTrCP1 and followed by an Alexa Fluor 555- or 488-conjugated secondary Abs, respectively, and fluorescence microscopy. DAPI was used to show the nuclei, and co-localizations of FOXO3 with βTrCP1 images are shown as the merged yellow/orange images. (B, C) Overexpression of βTrCP1-myc in MCF-7 cells (B) and 293T cells (C) confirms the subcellular localizations of βTrCP1-myc. At 48 hours after transfection, cells were fixed and stained with Abs against myc-tag or βTrCP1, a specific Ab that was developed by K. Strebel at NIH as described previously [22], and followed by an Alexa Fluor 647- or 488-conjugated secondary Abs, respectively, and fluorescence microscopy as described above. (3.00 MB TIF) [file pone.0011171.s006.tif]

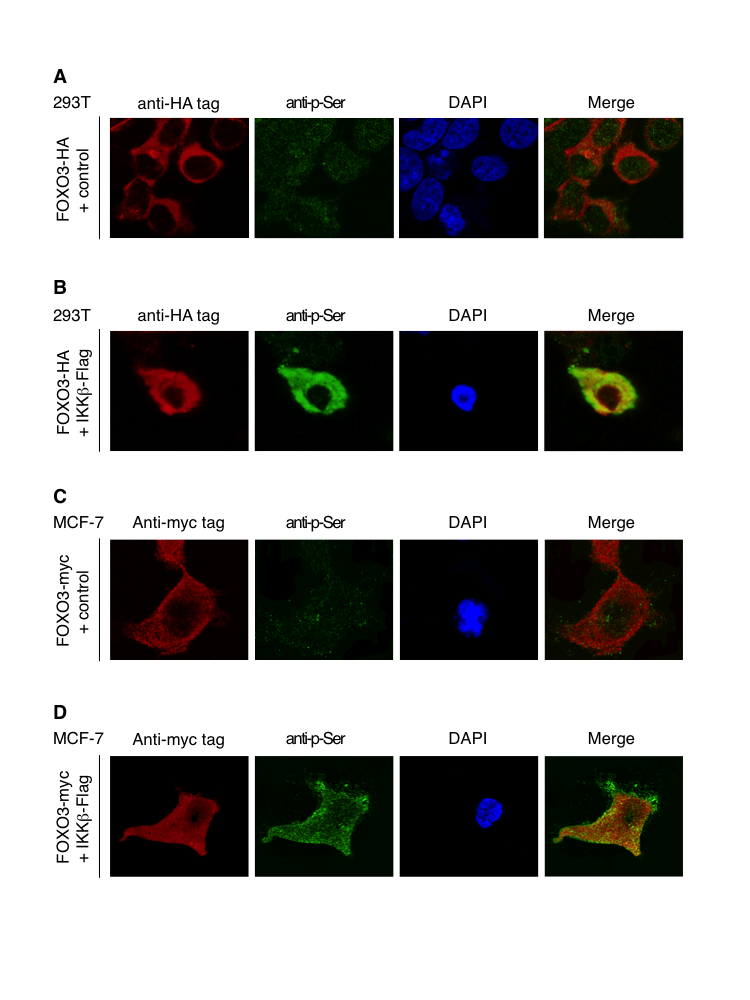

Supplement: Figure S7 — The phosphorylation status of FOXO3 protein in cells over-expressing FOXO3 and IKKβ. (A, B) 293T cells were co-transfected with FOXO3-HA vector plus control (A) or IKKβ-Flag (B) vector. Forty-eight hours after transfection, cells were washed, fixed, and stained with antibodies against HA-tag and phosphoserine (p-Ser) and followed by the Alexa Fluor 647 (red)- and Alexa Fluor 488 (green)-conjugated secondary antibodies, respectively, and fluorescence microscopy. DAPI was used to show the nuclei. Co-localizations between FOXO3-HA and p-Ser images are shown as the merged yellow/orange images. (C, D) MCF-7 cells were co-transfected with FOXO3-myc vector plus control (C) or IKKβ-Flag (D) vector. Forty-eight hours after transfection, cells were stained with antibodies against myc-tag and p-Ser and followed by secondary antibodies and fluorescence microscopy as described above. Co-localizations between FOXO3-myc and p-Ser images are shown as described. (3.00 MB TIF) [file pone.0011171.s007.tif]

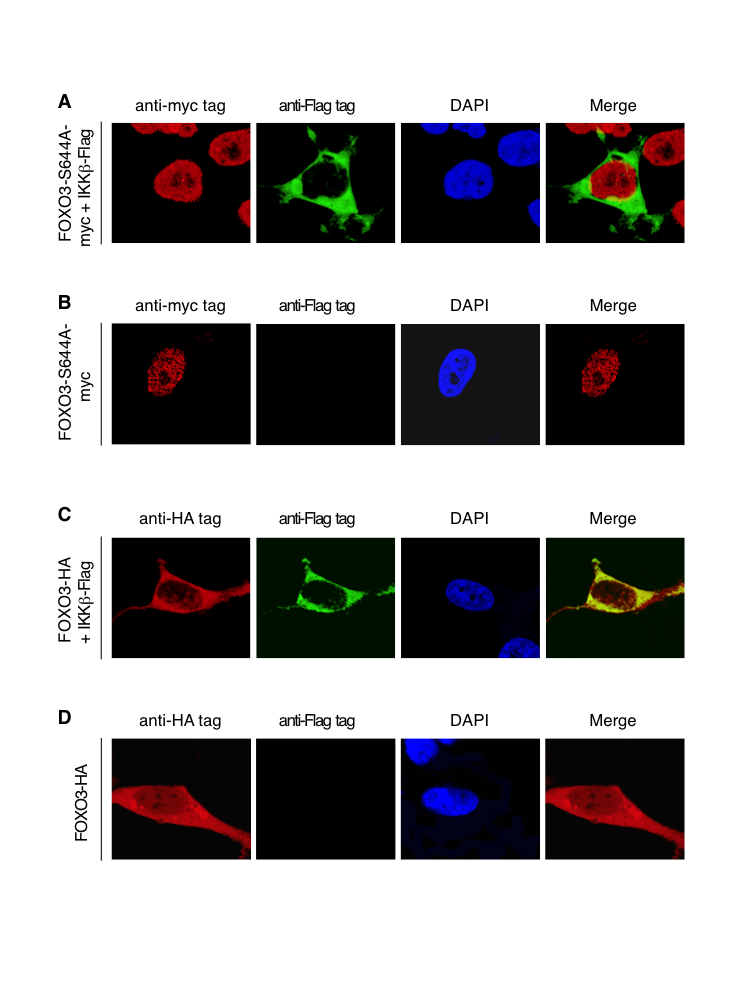

Supplement: Figure S8 — The subcellular localization of mutant FOXO3-S644A protein in cells overexpressed FOXO3-S644A and IKKβ. (A, B) 293T cells were co-transfected with mutant FOXO3-S644A-myc plus IKKβ-Flag (A) vectors or FOXO3-S644A-myc alone (B). Forty-eight hours after transfection, cells were washed, fixed, and stained with antibodies (Abs) against myc-tag and Flag-tag and followed by the Alexa Fluor 546 (red)- and Alexa Fluor 488 (green)-conjugated secondary Abs, respectively, and fluorescence microscopy. DAPI was used to show the nuclei. (C, D) MCF-7 cells were co-transfected with wild-type FOXO3-HA plus IKKβ-Flag vectors (C) or FOXO3-HA alone (D). At 48 hours post transfection, cells were fixed and stained with Abs against myc-tag and Flag-tag and followed by secondary Abs and fluorescence microscopy as described above. Co-localizations between FOXO3-HA and IKKβ-Flag images are shown as the merged yellow/orange images. (3.00 MB TIF) [file pone.0011171.s008.tif]

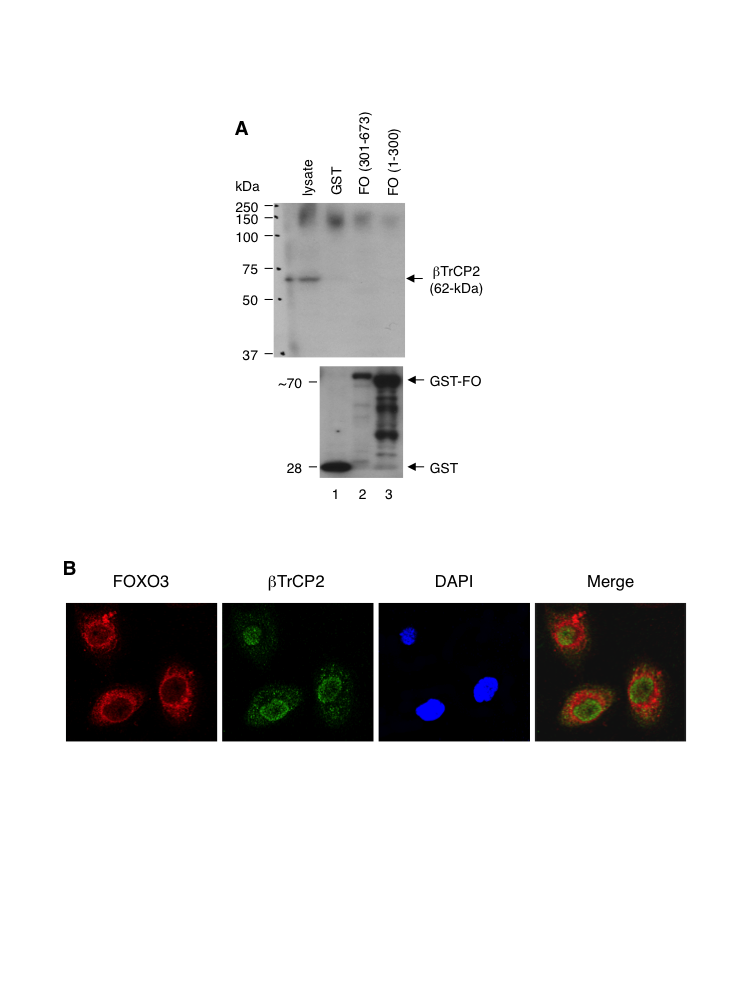

Supplement: Figure S9 — βTrCP2 does not bind FOXO3 protein significantly in vitro and in vivo. (A) GST-pull down in vitro assays. Whole lysates from 293T cells overexpression of IKKβ were incubated with the GST-FOXO3 [GST-FO (1–300) and GST-FO (301–673)] fusion proteins as indicated and GST alone (negative control), and analyzed by SDS-PAGE and immunoblotting with an anti-βTrCP2 antibody (Ab) (upper panel) and an anti-GST Ab (lower panel) as protein controls. (B) Co-localization between endogenous βTrCP2 and FOXO3 in HeLa cells. Cells cultured under normal conditions were stained with Abs against FOXO3 and βTrCP2 and followed by an Alexa Fluor 594 (red)- and Alexa Fluor 488 (green)-conjugated secondary Abs, respectively, and fluorescence microscopy. DAPI was used to show the nuclei. No significant co-localization (the merged yellow images) between endogenous FOXO3 with βTrCP2 was detected. (3.00 MB TIF) [file pone.0011171.s009.tif]
